# Impact of COVID-19 on patient–doctor interaction in a complex radiation therapy facility

**DOI:** 10.1007/s00520-020-05793-3

**Published:** 2020-10-02

**Authors:** Isacco Desideri, G. Francolini, L. P. Ciccone, G. Stocchi, V. Salvestrini, M. Aquilano, D. Greto, P. Bonomo, I. Meattini, V. Scotti, S. Scoccianti, G. Simontacchi, L. Livi

**Affiliations:** 1 Department of Biomedical, Experimental, and Clinical Sciences “Mario Serio”, University of Florence, Viale Morgagni 85, 50134 Florence, Italy; 2 Radiation Oncology Unit, University of Florence, Florence, Italy; 3 CyberKnife Center, Istituto Fiorentino di Cura ed Assistenza, Florence, Italy; 4 Radiation Oncology Unit, Azienda Ospedaliero-Universitaria Careggi, Florence, Italy

**Keywords:** COVID-19, Radiotherapy, QoL, Patient satisfaction

## Abstract

**Purpose:**

In the last months, Italy faced a COVID-19 emergency and implemented preventive measures in order to protect patients and healthcare providers from a disease outbreak. The pandemic control strategies impacted patient experience directly. Questionnaires evaluating patients reported measures (PREMs) may assess critical issues and represent a helpful tool to measure the patient perception of healthcare service. Our aim was to prospectively assess patient satisfaction about doctor–patient interaction in a high-volume radiation therapy and oncology center during the COVID-19 pandemic.

**Methods:**

Cancer patients receiving either systemic and/or radiation treatment underwent a survey. Two validated questionnaires (EORTC QLQ-C30, FACIT-TS-G version 1) and 14 specific questions evaluating patients’ perception of COVID-19 measures were administered.

**Results:**

One hundred twenty-five patients admitted to our department from 1–30 April 2020 completed the questionnaires. The majority (66.4%) of patients were women and the most common disease was breast cancer (40%). The average Global Health Status (GHS) of EORTC QLQ-C30 was 61.67. Emotional functioning, social, and cognitive domains obtained scores of 75.48, 80.13, and 84.67, respectively. FACIT-TS-G results revealed 120 patients rated the treatments effective and 108 patients thought the side effects were the same as expected or better. Most (89.6%) rated their treatment good, very good, or excellent. Concerning COVID-19-related questions, patients reported overall very good level of information.

**Conclusions:**

Despite the introduction of strict COVID-19 control measures, there was a high level of cancer outpatient satisfaction. The satisfaction levels may influence compliance, continuity of treatments, and patient–doctor communication, impacting the quality of clinical care in the next phases of the pandemic.

## Background

On March 11, 2020, the WHO Director-General confirmed the coronavirus (COVID-19) disease a pandemic with a very high case-fatality rate. After the outbreak of the COVID-19 in China, Europe, and the USA became the most affected regions [1, 2]. The majority of COVID-19 infections are characterized by mild symptoms such as temperature ≥ 37.8 °C, cough, ageusia, and anosmia [3]. However, there is a high risk of severe pulmonary infection and death, particularly for the elderly and populations with comorbidities [4, 5]. In Italy, COVID-19 has led to severe overloading on the national healthcare system and consequently prompted a series of containment measures impacting healthcare services. On March 8, the Italian Government implemented extraordinary hospital measures aiming to decrease the viral transmission from patient to patient and to health professionals [6]. This involved temperature check and hand disinfection before all departments admittance and use of personal protective equipment (PPE) by health professionals and patients. The hospital also adopted a “no visitor” rule and limited caregivers’ access to hospitals. Most departments also minimized the healthcare workforce, aiming to reduce the risk of COVID-19 transmission. Additionally, many routine medical examinations were deferred or replaced by telephone consultation. In particular, cancer patients represent a frail population reported to be at increased risk of symptomatic presentation and death [7]. Thus, preventive measures appear of utmost importance in this setting. However, pandemic control strategies also impact patient experience directly. In these circumstances, an optimal patient–doctor interaction remains mandatory particularly to support cancer patients. Levels of satisfaction may influence compliance, continuity of treatments, and patient–doctor communication improving the overall quality of clinical care [8]. Questionnaires evaluating patient-reported measures (PREMs) may assess critical issues and represent a helpful tool to measure the patient perception of healthcare service [9, 10]. Hence, the aim of this study was to prospectively assess patient satisfaction about doctor–patient interaction in high-volume radiation therapy and oncology center during the COVID-19 pandemic. The information collected may allow us to evaluate how a cancer patient’s perspective is affected by the outbreak of COVID-19 disease and consequent strategies of infection control.

## Methods

### Population, study design

The present study was conducted at the Radiation Oncology Unit of the Careggi University Hospital of Florence, Italy. This is a prospective monocenter study including cancer patients admitted to the department to receive either systemic and/or radiation treatment. All patients recruited underwent a survey approved by our institutional ethics review board.

Two validated questionnaires (EORTC QLQ-C30, FACIT-TS-G version 1) [11, 12] were administered to the recruited outpatients. Additionally, an internally developed survey consisting of 14 questions evaluating patients’ perception of COVID-19 measures was administered. To be eligible, cancer patients had to be aged 18 years or older without cognitive impairment. Patients anonymously filled in the Italian version of the questionnaires at their hospital access. Both patients accessing our facility at treatment start and during their treatment were included. Patients were asked to deposit completed questionnaires into a closed survey box placed in the patient waiting area. Only one form was filled by each included patient.

The aim of the present study was to evaluate Health-Related Quality of Life (HRQoL), patient satisfaction, and level of patient knowledge and satisfaction with COVID-19 precautions.

### Quality of life analysis

EORTC QLQ-C30 was analyzed according to the scoring manual and a linear transformation of results on a 0 to 100 scale was performed. Scores for each of the following domains were assessed: Global Health Status (GHS), Functional Scales (Physical, Role, Emotional, Cognitive and Social Functioning), and Symptom Scales (Fatigue, Nausea, Pain, Dyspnea, Insomnia, Appetite Loss, Constipation, Diarrhea, Financial Difficulties).

FACIT-TS-G results were reported in terms of the percentage of patients satisfied with the healthcare service provided. A specific survey regarding COVID-19 preventive measures was internally developed on the basis of a pre-existent questionnaire used during the 2003 SARS outbreak [13].

Our survey consisted of two main subgroups: patient’s level of information about the pandemic (5 questions) and patient’s level of satisfaction for health-related measures during the pandemic (9 questions), as shown in Table 4. Patients were asked to report their agreement regarding each different statement (strongly disagree, disagree, agree, strongly agree). Patients’ demographic information (sex, age range, level of education) and primary tumor diagnosis were also collected. Descriptive statistics was performed to report analysis results. MedCalc Software version 19.2.1 was employed for the statistical analysis. Questionnaires with missing data were not included in the analysis.

## Results

Overall, complete data from 125 patients admitted to our department from 1 to 30 April 2020 were collected and analyzed. The complete cohort was mainly women (66.4%), the median age range was between 61 and 75 years, and the most common disease was breast cancer (40%). Undergraduate level of education was the most represented (78.4%). Further details of patients and tumor characteristics are provided in Table 1.

**Table 1:** Patients’ characteristics

	*n*	%
Sex	41 males	32.8
	83 females	66.4
	1 unknown	0.8
Age (years)	2 (18–30)	1.6
	20 (31–45)	16
	39 (46–60)	31.2
	48 (61–75)	38.4
	14 (76–90)	11.2
	1 (> 90)	0.8
	1 unknown	0.8
Median age (range)	61–75	
Level of education	15 elementary school	12
	37 middle school	29.6
	46 high school	36.8
	26 graduate–post-graduate	20.8
	1 unknown	0.8
Primary malignancy	50 breast	40
	17 lung	13.6
	8 genito-urinary	6.4
	3 gastro-intestinal	2.4
	7 Gynecological	5.6
	10 head and neck	8
	10 brain	8
	4 hematologic	3.2
	16 other	12.8

**Fig 1:**
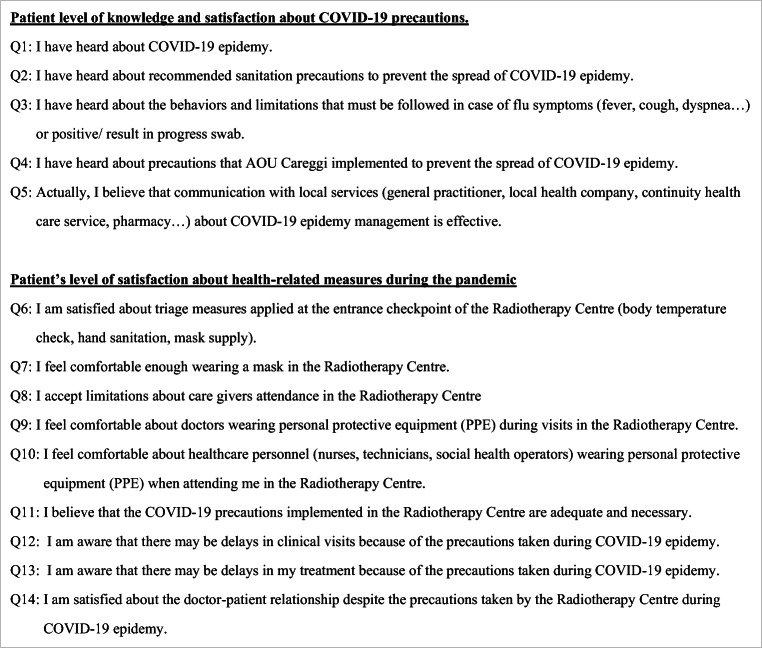
Average scores for specific subdomains of EORTC QLQ-C30 questionnaires

The results of EORTC QLQ-C30 showed an average GHS of 61.67. Among functional domains, emotional functioning obtained the lowest score (75.48), while the social and cognitive domains were minimally impaired (80.13 and 84.67, respectively). Fatigue and insomnia were the most reported symptoms, with average scores of 34.93 and 32.27, respectively. Full results are reported in Fig. 1.

According to the results of the FACIT-TS-G questionnaire, 120 patients (96.4%) rated the treatments effective and 108 patients (86.4%) thought the side effects were the same as expected or better. Eighty-four patients (67.4%) felt the doctors helped them evaluate the effects of their treatments, 102 patients (81.6%) stated they received the right treatment for them, and 87 (69.6%) felt satisfied with the ongoing treatment. Ninety-eight patients (78.4%) would recommend their treatment to other patients and 97 (77.6%) would choose the same treatment again. One hundred twelve patients (89.6%) rated their treatment good, very good, or excellent. FACIT-TS-G results are summarized in Table 2.

**Table 2:** FACIT-TS-G results

	**A lot worse**	**A little worse**	**About the same**	**A little better**	**A lot better**
TS1: Compared to what you expected, how do you rate the effectiveness of the treatment so far?	2 (1.6%)	2 (1.6%)	53 (42.4%)	38 (30.4%)	30 (24%)
TS2: Compared to what you expected, how do you rate the side effects of treatment so far?	3 (2.4%)	14 (11.2%)	41 (32.8%)	42 (33.6%)	25 (20%)
	**No, not at all**	**Yes, to some extent**	**Yes, for the most part**	**Yes, Completely**	
TS3: Did your doctor(s) help you evaluate the effects of your treatment so far?	9 (7.2%)	32 (25.6%)	56 (44.8%)	28 (22.4%)	
TS4: Do you feel you received the treatment that was right for you?	1 (0.8%)	22 (17.6%)	60 (48%)	42 (33.6%)	
TS5: Are you satisfied with the effects of this treatment so far?	2 (1.6%)	36 (28.8%)	52 (41.6%)	35 (28%)	
	**No**	**Maybe**	**Yes**		
TS6: Would you recommend this treatment to others with your illness?	3 (2.4%)	25 (20%)	97 (77.6%)		
TS7: Would you choose this treatment again?	3 (2.4%)	25 (20%)	97 (77.6%)		
	**Poor**	**Fair**	**Good**	**Very Good**	**Excellent**
TS8: How do you rate this treatment overall?	1 (0.8%)	12 (9.6%)	44 (35.2%)	40 (32%)	28 (22.4%)

Concerning COVID-19-related questions, patients reported overall very good level of information regarding the required procedures to optimize patient safety and minimize disease spread. In particular, 116 (92.8%), 121 (96.8%), and 120 (96%) patients felt informed about their disease, infection control requirements (e.g., wearing a mask, limiting visitors), and limitations in case of contact with a positive patient or positive swab, respectively. Moreover, 110 (88%) and 97 (77.6%) patients were well informed regarding department measures to prevent the spread of the disease and rated communication as effective with the healthcare systems/operators. The complete results are listed in Table 3.

**Table 3:** COVID-19 focused questionnaire results

	Category of response (*n*, %)			
	Strongly disagree	Disagree	Agree	Strongly agree
Patient level of knowledge and satisfaction about COVID-19 precautions				
Q1: I have heard about COVID-19 epidemy	2 (1.6%)	7 (5.6%)	75 (60%)	41 (32.8%)
Q2: I have heard about recommended sanitation precautions to prevent the spread of COVID-19 epidemy.	3 (2.4%)	1 (0.8%)	40 (32%)	81 (64.8%)
Q3: I have heard about the behaviors and limitations that must be followed in case of flu symptoms (fever, cough, dyspnea…) or positive/result in progress swab.	1 (0.8%)	4 (3.2%)	41 (32.8%)	79 (62.2%)
Q4: I have heard about precautions that AOU Careggi implemented to prevent the spread of COVID-19 epidemy.	4 (3.2%)	11 (8.8%)	58 (46.4%)	52 (41.6%)
Q5: Actually, I believe that communication with local services (general practitioner, local health company, continuity health care service, pharmacy…) about COVID-19 epidemy management is effective.	4 (3.2%)	24 (19.2%)	64 (51.2%)	33 (26.4%)
Patient’s level of satisfaction about health-related measures during the pandemic				
Q6: I am satisfied about triage measures applied at the entrance checkpoint of the Radiotherapy Centre (body temperature check, hand sanitation, mask supply).	1 (0.8%)	1 (0.8%)	40 (32%)	83 (66.4%)
Q7: I feel comfortable enough wearing a mask in the Radiotherapy Centre.	0	1 (0.8%)	33 (26.4%)	91 (72.8%)
Q8: I accept limitations about care givers attendance in the Radiotherapy Centre	4 (3.2%)	5 (4%)	33 (26.4%)	83 (66.4%)
Q9: I feel comfortable about doctors wearing personal protective equipment (PPE) during visits in the Radiotherapy Centre.	0	4 (3.2%)	34 (27.2%)	87 (69.6%)
Q10: I feel comfortable about healthcare personnel (nurses, technicians, social health operators) wearing personal protective equipment (PPE) when attending me in the Radiotherapy Centre.	0	3 (2.4%)	35 (28%)	87 (69.6%)
Q11: I believe that the COVID-19 precautions implemented in the Radiotherapy Centre are adequate and necessary.	1 (0.8%)	0	38 (30.4%)	86 (68.8%)
Q12: I am aware that there may be delays in clinical visits because of the precautions taken during COVID-19 epidemy.	4 (3.2%)	6 (4.8%)	48 (38.4%)	67 (53.6%)
Q13: I am aware that there may be delays in my treatment because of the precautions taken during COVID-19 epidemy	3 (2.4%)	13 (10.4%)	46 (36.8%)	63 (50.4%)
Q14: I am satisfied about the doctor-patient relationship despite the precautions taken by the Radiotherapy Centre during COVID-19 epidemy	3 (2.4%)	7 (5.6%)	44 (35.2%)	71 (56.8%)

## Discussion

Cancer management during the COVID-19 pandemic is one of the most important issues for clinical oncologists [14]. Extraordinary measures introduced during a public health emergency may alter patients’ HRQoL and satisfaction level, and previous reports have already shown that patients’ compliance and treatment outcomes are significantly influenced by doctor–patient interaction [8, 15]. In the University Hospital of Florence, the strategies of pandemic containment have led to a reorganization of routine clinical activity, including oncology departments. The current study reports information from cancer patients receiving either systemic and/or radiation treatment in palliative, curative, neoadjuvant, and adjuvant settings. Commonly, the cancer population is vulnerable and worried about delay and interruption of anticancer treatments. Patients’ perception may be also affected by many aspects (e.g., healthcare services, hospital environment, support and communication from the healthcare team, and comprehension of therapeutic strategy). In particular, worse interaction with healthcare workers may negatively impact compliance and the tendency to ask for help. It may decrease patients’ satisfaction with care and consequently influence their HRQoL [13]. The EORTC QLQ-C30 questionnaire is a validated and reliable tool to measure cancer patients’ HRQoL, commonly used in clinical trials and prospective evaluations [11]. Similarly, FACIT-TS-G explores multiple aspects of care, including patient–doctor interaction and overall patient experience of treatment [16]. All EORTC QLQ-C30 domains showed overall good HRQoL. Interestingly, high GHS scores were reported (61.67 points), despite the extraordinary measures strictly adopted in our institution. Indeed, breast cancer was the most common disease in the analyzed cohort, and previously published experiences about breast cancer patients (> 50 years) reported comparable EORTC QLQ-C30 GHS score (65.16) [17]. Thus, indirect comparison with routine cancer management suggests that preventive measures had a limited impact on patients HRQoL. Furthermore, FACIT-TS-G questionnaires showed that most of the patients were satisfied with the healthcare service provided. In addition, aiming to explore general levels of information and satisfaction about COVID-19 preventive measures, we administered a specific survey. Most of the patients reported they felt well informed about COVID-19 disease and consequent strategies of infection control. These results suggest that patient satisfaction in our department was minimally influenced by COVID-19 preventive measures.

Moreover, measures adopted during the COVID-19 outbreak seem to have a limited influence on clinical activity and workload burden of our department [18]. Notably, Q5 was the only statement with approximately 20% of patients expressing a low agreement level. However, this statement was mainly related to an opinion about outpatients’ home care, and this result could represent patients’ perception of local assistance rather than low compliance to specific measures implemented for the COVID-19 pandemic within our facility. To our knowledge, evidence related to cancer patients’ perspective and satisfaction during COVID-19 is limited. However, our findings are in line with the experience published after the severe acute respiratory syndrome (SARS) outbreak. As part of this, a survey in 2007 compared the experience during the outbreak with a period following the cessation of SARS control measures. The study highlighted that the overall level of satisfaction was similar during and after the outbreak of SARS, suggesting a higher compliance for patient–doctor interaction issues during emergency circumstances [19]. Similarly, Tang et al. assessed how SARS control strategies impacted patient experience and influenced satisfaction in 149 cancer patients. Despite the implementation of infection control measures, 94% of patients were satisfied with doctor–patient interaction in this study [13]. In line with previous experience, the majority of patients in our study were satisfied regarding the introduction of infection control strategies with a high confidence in the healthcare team. During the COVID-19 outbreak, the Italian government reported the daily updates of the COVID-19 situation worldwide, recommending the preventive measures to control transmission. Moreover, media campaigns described doctors and healthcare team as “heroes” in the frontline, contributing to preserve patients’ satisfaction and increase compliance during the public health emergency. In addition, our department adopted COVID-19 telephone triage for outpatients, teleconsultation, and oral chemotherapy home delivery. These clinic service delivery modifications may have played an important role in maintaining a high level of satisfaction in patient–healthcare team interaction. According to our experience, patients had also a positive perception of hospital precautions and felt confident in doctors’ recommendations. Despite the fear of infection widely reported in cancer patients [20], the high level of satisfaction supports effective patient–doctor communication and valid assistance from the healthcare team in our center. Thus, our results suggest the importance of optimal patient and caregiver education programs during emergency circumstances (Table 4).

**Table 4:** English translation of COVID-19-focused questionnaire

**Patient level of knowledge and satisfaction about COVID-19 precautions**	
Q1: I have heard about COVID-19 epidemy.	
Q2: I have heard about recommended sanitation precautions to prevent the spread of COVID-19 epidemy.	
Q3: I have heard about the behaviors and limitations that must be followed in case of flu symptoms (fever, cough, dyspnea…) or positive/ result in progress swab.	
Q4: I have heard about precautions that AOU Careggi implemented to prevent the spread of COVID-19 epidemy.	
Q5: Actually, I believe that communication with local services (general practitioner, local health company, continuity health	
care service, pharmacy…) about COVID-19 epidemy management is effective.	
**Patient’s level of satisfaction about health-related measures during the pandemic**	
Q6: I am satisfied about triage measures applied at the entrance checkpoint of the Radiotherapy Centre (body temperature check, hand sanitation, mask supply).	
Q7: I feel comfortable enough wearing a mask in the Radiotherapy Centre.	
Q8: I accept limitations about care givers attendance in the Radiotherapy Centre	
Q9: I feel comfortable about doctors wearing personal protective equipment (PPE) during visits in the Radiotherapy Centre.	
Q10: I feel comfortable about healthcare personnel (nurses, technicians, social health operators) wearing personal protective equipment (PPE) when attending me in the Radiotherapy Centre.	
Q11: I believe that the COVID-19 precautions implemented in the Radiotherapy Centre are adequate and necessary.	
Q12: I am aware that there may be delays in clinical visits because of the precautions taken during COVID-19 epidemy.	
Q13: I am aware that there may be delays in my treatment because of the precautions taken during COVID-19 epidemy.	
Q14: I am satisfied about the doctor-patient relationship despite the precautions taken by the Radiotherapy Centre during COVID-19 epidemy.	

In conclusion, there was a high level of cancer outpatient satisfaction, despite the introduction of strict COVID-19 control measures in our radiation therapy and oncology department. Patients’ satisfaction questionnaires are relevant instruments, especially in the cancer patients’ population. High satisfaction levels may influence compliance, continuity of treatments, and patient–doctor communication, improving the quality of clinical care in the next phases of the pandemic.

### Availability of data and material

Not applicable.
